# Efficacy of percutaneous kyphoplasty on vertebral compression fractures with different bone mineral densities: a retrospective study

**DOI:** 10.1186/s12891-023-06341-w

**Published:** 2023-04-10

**Authors:** Chen Ge, Zhe Chen, Peng Cao

**Affiliations:** Department of Orthopedics, Shanghai Ruijin Hospital, Shanghai Jiao Tong University School of Medicine, No. 999, Xiwang road, Jiading district, Shanghai, 201801 China

**Keywords:** Percutaneous kyphoplasty, Vertebral compression fractures, Bone mineral density, Wedge angle, Kyphotic angle

## Abstract

**Background:**

This study was performed to investigate the clinical efficacy of percutaneous kyphoplasty (PKP) for vertebral compression fractures with different bone mineral densities (BMD).

**Methods:**

We performed a retrospective analysis of 232 patients with single-segment vertebral compression fractures who underwent PKP. Patients were divided into the normal BMD, osteopenia, and osteoporosis groups according to their average lumbar BMD before surgery. The visual analog scale (VAS) was used to compare differences in pain relief before and after surgery in each group. Corrections of the wedge angle and kyphotic angle before and after surgery were observed using anteroposterior and lateral radiographs and compared among the groups, as was the incidence of bone cement leakage.

**Results:**

Patients were followed up for 6–12 months, with an average follow-up time of 9.12 ± 1.68 months. The VAS score, wedge angle, and kyphotic angle of the three groups of patients decreased significantly at the end of the follow-up (*P* < 0.05). The changes in VAS score and wedge angle correction in the osteoporosis group were significantly larger than those in the normal BMD and osteopenia groups (*P* < 0.05). There were no significant differences among the three groups in terms of kyphotic angle correction or bone cement leakage rates (*P* > 0.05).

**Conclusions:**

PKP has a positive effect on vertebral compression fractures with different BMD, and is especially suitable for osteoporotic vertebral compression fractures.

## Background

Osteoporosis is a common disease in elderly patients, which can lead to increased fragility and a propensity for fracture [1, 2]. According to epidemiological investigations, the incidence of senile vertebral compression fractures in patients aged > 70 years is approximately 20%, while the rate in postmenopausal women is approximately 16% [3, 4]. As the world population continues to age, the morbidity of osteoporosis is increasing annually, becoming a major health problem worldwide [5]. Vertebral compression fracture, especially osteoporotic vertebral compression fracture, is a significant complication of osteoporosis [6]. Vertebral compression fractures can lead to chronic low back pain, kyphosis, posture restriction, and secondary fatigue of the back muscles, which all can seriously affect the quality of life and physical and mental health of patients [7]. Therefore, prevention of deformity progression and correction of the existing deformity are important for this population.

In addition to traditional conservative treatments, percutaneous kyphoplasty (PKP) has been widely clinically used for the treatment of osteoporotic vertebral compression fractures and has proven to be effective and safe [8–10]. This surgery can quickly relieve a patient’s pain, restore the geometric deformity of the compressed vertebral body, and avoid long-term posture restriction and low back muscle fatigue [11]. The indications for PKP are not limited to osteoporotic vertebral compression fractures; this technique can also be utilized to treat vertebral compression fractures with a slight decrease in or normal bone mineral density (BMD) [7, 12]. However, there have been relatively few comparative analyses on the efficacy of PKP in treating vertebral compression fractures with different BMD. Therefore, this study aimed to compare the therapeutic effect of PKP on vertebral compression fractures with different BMD using retrospective data.

## Methods

### Patients

A total of 232 patients who received PKP treatment in our hospital for single-segment vertebral compression fractures between June 2017 and January 2020 were retrospectively analyzed. The inclusion criteria were as follows: (1) patients aged ≥ 55 years without a history of high-energy trauma; (2) a main complaint of chronic low back pain and poor pain relief after a period of conservative treatment, such as taking drugs and physical therapy; (3) lateral X-rays of the thoracic and lumbar spine indicated a single-segment vertebral compression fracture between the 12 thoracic and two lumbar vertebrae, manifesting as a geometrical deformity of the vertebral body, and physical examination showed obvious lumbar spinous process tenderness and/or percussive pain in the compression segment of the vertebrae; and (4) CT or MRI showed a fresh fracture or a fracture that had not fully healed in the vertebral body. The exclusion criteria were as follows: (1) young and middle-aged patients; (2) a clear history of high-energy violent trauma; (3) serious burst fractures, rupture of the posterior edge of the vertebral body, and fracture fragments occupying the space in the spinal canal; (4) severe damage to the middle and posterior columns during fracture, causing spinal instability and requiring decompression or posterior implant fixation; and (5) pathological vertebral fractures caused by suspected benign or malignant tumors or other factors.

Before the operation, the lumbar spine BMD of all patients was measured with dual-energy X-ray absorptiometry. The average T-score of the lumbar spine was subsequently calculated. Patients were divided into the normal bone mass group (lumbar spine T value greater than − 1), osteopenia group (lumbar spine T value between − 1 and − 2.5), and osteoporosis group (lumbar spine T value is less than − 2.5) according to the WHO classification.

All methods were performed in accordance with the Declaration of Helsinki. The study was approved by the ethics committee of the Shanghai Ruijin Hospital, Shanghai Jiao Tong University School of Medicine. Informed consent was obtained from all participants.

### Surgery method

All patients received preoperative intramuscular injections of a capsulopoly. The patients were treated with unilateral pedicle punctures under local anesthesia. The patient was placed in the prone position to undergo fluoroscopy, and the injured vertebral segment was confirmed under fluoroscopy using a C-arm X-ray machine. The pedicle projection position on the body surface is marked. The vertebral body was reduced with a spinal surgical stent in the hyperextension position before the bone cement puncture. After routine disinfection and draping, a percutaneous needle was used to puncture the vertebral body under the guidance of a C-arm machine. The puncture needle on the anterior radiograph was located in the upper quadrant of the pedicle projection, and the puncture depth was observed on the lateral radiograph to reach the anterior 1/2 to 2/3 of the vertebral body. After the working channel was established, the balloon retractor was pushed to the front 3/4 position of the vertebral body under C-arm machine monitoring, and the pressure was maintained below 250 PSI. The vertebral body was subsequently reduced, and polymethyl methacrylate bone cement mixed with a contrast agent was prepared and injected into the vertebral body through the puncture working channel. Bone cement injection into the vertebral body was closely monitored using a C-arm machine to avoid leakage. Patients were provided basic treatment with calcium and vitamin D3 after surgery, as well as anti-osteoporotic symptomatic therapy with alendronate sodium or calcitriol. If the patient still experienced pain, adjunctive symptomatic etoricoxib treatment was provided. Bone cement was filled in the front 3/4 of the vertebral body. After the operation, the patients were routinely bedridden for 1–2 days and underwent follow-up for 6–12 months.

#### Pain assessment

The visual analog scale (VAS) was used to quantify the patient’s low back pain before the operation and at the end of the follow-up, and the changes in the VAS score was calculated.

#### Morphology measurement of the vertebral body

The morphology of the injured vertebral body was assessed preoperatively and during follow-up using frontal and lateral radiographs of the lumbar spine. To evaluate the morphology of the compressed vertebral body, the wedge angle (WA) of the vertebral body was measured based on the lateral radiograph of the lumbar spine, which is the angle between the two lines connecting the upper and lower endplates of the vertebral body [13]. Changes in WA before and after surgery were calculated. The kyphotic angle (KA) of the three consecutive vertebrae containing the injured vertebra was used to evaluate the correction of the entire kyphotic deformity before and after surgery, which was defined as the angle between the line connecting the upper endplate of the uppermost vertebra and the line connecting the lower endplate of the last vertebra in the three consecutive vertebrae [13].

The bone cement leakage rates in each group were calculated by observing the presence of bone cement leakage based on anterior and lateral lumbar spine X-rays during follow-up.

### Statistical analysis

All statistical analyses were performed using the SPSS software (version 21.0; IBM Corporation, USA). Shapiro-Wilk test was used to test the normality of data. Continuous variables were expressed as mean ± standard deviation in cases of normal distribution, and as median and interquartile range in cases of non-normal distribution. Normally distributed data were compared among groups using one-way ANOVA, and non-normally distributed data were compared among groups using Kruskal-Wallis test. Categorical variables are expressed as percentages, and Fisher’s test was used to compare the differences among the three groups. A *P*-value < 0.05 indicated a significant difference.

## Results

The demographic characteristics of the enrolled patients are shown in Table 1. Overall, there were 64 patients in the normal BMD group, 81 in the osteopenia group, and 87 in the osteoporosis group. The patients ranged in age from to 56–88 years. No significant differences were found among the three groups in terms of age, sex, or bone cement injection volume before surgery (Table 1). All patients were followed for 6–12 months, and there was no statistical difference in the follow-up time between the groups. No serious complications, such as pulmonary embolism, venous embolism, or intraspinal leakage of bone cement after surgery, were observed.

**Table 1:** Comparison of demographic characteristics of patients before surgery in each group

	Normal BMD group (n = 64)	Osteopenia group (n = 81)	Osteoporosis group (n = 87)	*F/X* ^*2*^	*P*
Age (years)	65.35 ± 7.43	66.31 ± 8.87	68.56 ± 8.62	1.08*	0.35
Sex (Male/Female)	16/48	18/63	18/69	0.11#	0.94
BMD (T-score)	0.22 ± 0.067	-1.68 ± 0.33	-3.07 ± 0.48	0.63*	0.031
Number of different operative locations (T12/L1/L2)	16/32/16	27/39/15	33/30/24	2.12#	0.71
Volume of bone cement	5.20 ± 1.16	4.78 ± 1.88	5.71 ± 1.53	0.07*	0.93
Surgery time (min)	15.92 ± 2.36	15.64 ± 2.50	15.39 ± 2.58	0.02*	0.12
Follow-up time (month)	9.15 ± 1.03	9.19 ± 1.62	9.47 ± 1.58	0.19*	0.82

Continuous variables were expressed as mean ± standard deviation, and compared by one-way ANOVA. Categorical variables were expressed as percentages, and Fisher’s test was used to compare the difference among three groups. * represents F value while # indicates *X*^*2*^. BMD, bone mineral density; T12: the 12th thoracic vertebra; L1: the 1st lumbar vertebra; L2: the 2nd lumbar vertebra

The VAS scores for lower back pain in the three groups were all significantly lower after surgery compared to those before surgery (*P* < 0.001, Table 2). The VAS of the normal BMD group was reduced from 7.57 ± 1.24 preoperatively to 2.87 (2.25–3.74) postoperatively, that of the osteopenia group was reduced from 8.24 ± 1.21 preoperatively to 3.54 (2.44–4.59) postoperatively, and that of the osteoporosis group was reduced from 8.57 ± 1.15 preoperatively to 2.94 (2.15–3.84) postoperatively. Among the three groups, the change in VAS score in the osteoporosis group was significantly larger than that in the normal BMD (*P* = 0.039) and osteopenia (*P* = 0.001) groups. There was no significant difference between the change in VAS score in the normal BMD group and that in the osteopenia group (*P* = 1.0).

**Table 2:** Comparison of the change of VAS, wedge angle and kyphosis angle in each group

	Normal BMD group (n = 64)	Osteopenia group (n = 81)	Osteoporosis group (n = 87)	*P*
Change of VAS after and before surgery	4.71 (4.15–5.17)	4.58 (3.91–5.24)	5.45 (5.00-5.95)	< 0.001
Change of WA after and before surgery (°)	4.34 ± 1.16	5.87 ± 1.93	9.78 ± 1.39	< 0.001
Change of KA after and before surgery (°)	2.78 ± 2.34	3.17 ± 1.52	2.38 ± 2.36	0.061

Shapiro-Wilk test was used to test the normality of data. Continuous variables were expressed as mean ± standard deviation in cases of normal distribution, and as median and interquartile range in cases of non-normal distribution. Normally distributed data were compared among groups using one-way ANOVA, and non-normally distributed data were compared among groups using Kruskal-Wallis test. BMD, bone mineral density; VAS, visual analogue scale; WA, wedged angle; KA, kyphotic angle

The WA in all three groups significantly decreased after surgery compared to that before surgery (*P* < 0.001, Table 2). The WA of the normal BMD group was reduced from 14.69 ± 1.31° preoperatively to 10.35 ± 1.65° postoperatively, that of the osteopenia group was reduced from 17.49 ± 1.73° preoperatively to 11.62 ± 1.94° postoperatively, and that of the osteoporosis group was reduced from 18.54 ± 2.57° preoperatively to 8.76 ± 1.35° postoperatively. Among the three groups, the change in WA in the osteoporosis group was significantly larger than that in the normal BMD group (*P* < 0.001) and the osteopenia group (*P* = 0.019). Furthermore, there was no significant difference between the change in WA in the normal BMD group and that in the osteopenia group (*P* = 0.071). This result indicates that PKP can effectively correct vertebral compression deformity in patients in the three groups and that the correction effect is better for patients with osteoporosis.

The KA in all three groups was significantly decreased after surgery compared to that before surgery (*P* < 0.001, Table 2). The KA of the normal BMD group was reduced from 11.69 ± 2.90° preoperatively to 8.91 ± 2.73° postoperatively, that of the osteopenia group was reduced from 13.41 ± 1.35° preoperatively to 10.24 ± 1.94° postoperatively, and that of the osteoporosis group was reduced from 17.18 ± 2.62° preoperatively to 14.80 ± 2.10° postoperatively. There were no significant differences among the three groups (*P* = 0.061).

The bone cement leakage rates in the normal BMD, osteopenia, and osteoporosis groups were 12.5% (8/64), 6.2% (5/81), and 16.1% (14/87), respectively. There was no statistical difference in bone cement leakage rate between the groups (*P* = 0.60), as shown in Table 3.

**Table 3:** Comparison of Bone Cement Leakage in Each Group

	Normal BMD group (n = 64)	Osteopenia group (n = 81)	Osteoporosis group (n = 87)	*X* ^*2*^	*P*
Number of bone cement leakage	8	5	14	1.37	0.60
Bone cement leakage rate	12.5%	6.2%	16.1%		

BMD, bone mineral density

## Discussion

A decrease in the strength and stiffness of the vertebral body after osteoporosis is an important reason for vertebral body compression fractures. PKP exerts a good therapeutic effect on various degrees of osteoporotic compression vertebral fractures [14, 15]. Zhou et al. further found that PKP is a safe and effective surgical method for the treatment of vertebral compression fractures [16]. In addition, Zhang et al. demonstrated that PKP can achieve satisfactory clinical efficacy in treating osteopenic thoracolumbar compression fractures [17]. Consistent with their results, our study showed that PKP can effectively relieve low back pain and correct geometric deformities in vertebral compression fractures with normal BMD, osteopenia, and osteoporosis. However, compared to patients with osteopenia and normal BMD, the effects of PKP on relieving pain and recovering vertebral morphology in patients with osteoporosis were more significant, and there was neither a significant difference in the bone cement leakage rate nor a significant advantage in the correction of kyphosis. These results suggest that PKP can be used in patients with vertebral compression fractures with different BMDs, especially in patients with osteoporosis.

Considerable in vivo and in vitro evidence has shown that BMD is an important factor affecting the postoperative efficacy of PKP in the treatment of vertebral compression fractures [18]. Biomechanical experimental studies have further confirmed that the lower the BMD of the vertebral body, the better the recovery of its strength and stiffness after bone cement filling. Heini et al. further observed that the augmentation of vertebral strength and stiffness after bone cement injection was negatively correlated with BMD [19]. Studies have also demonstrated the role of bone density in the biomechanical recovery of the vertebral body after bone cement filling [20–23]. The fact that the bone cement filling effect is better in patients with low BMD also explains why patients with osteoporosis obtained better correction of geometric deformity in our study.

Bone cement leakage following PKP is a common complication of this procedure due to the space formed by balloon kyphoplasty in the vertebral body. The bone cement leakage rate is reported to be 1–9% in PKP, which is significantly lower than the rate in patients treated with percutaneous vertebroplasty [24]. When bone cement leaks into the spinal canal or vein, it produces prolonged mechanical pressure and releases toxic substances, thereby leading to serious consequences [25]. Gao et al. demonstrated that the bone cement leakage rate was significantly increased in patients with low BMD who underwent PKP [26]. However, in this study, although the bone cement leakage rate in the osteoporosis group was slightly higher, there was no statistically significant difference compared to that in the other two groups. Therefore, in patients with osteoporotic vertebral compression fractures, the risk of bone cement leakage after PKP is not significantly increased.

The mechanisms of pain relief after PKP are as follows: (1) During the solidification process of bone cement, heat produces a certain burning effect on nerve endings, which relieves pain; (2) after a compression fracture, the axis of gravity of the body moves forward, and to maintain the balance of the body posture, the muscles of the lower back will be in a state of excessive contraction for a long time, resulting in chronic pain in the lower back [27]. PKP can relieve lower back pain by restoring the abnormal geometry of the vertebral body and stabilizing the abnormal biomechanics [28]. In addition to relieving pain, PKP can restore geometric deformities of the compressed vertebral body. The possible mechanism underlying this may involve the common tiny bone “cracks” in the compressed fractured vertebral body, which result in a certain micro-movement space inside the vertebral body. In the hyperextension position, the soft tissue around the injured vertebra can recover from the geometric deformity of the compressed vertebral body by pulling and distracting. Thereafter, the inserted endogenous balloon distractor further restores the geometric deformity of the vertebral body. Finally, re-collapse of the distracted vertebral body can be prevented by fixation with bone cement. In patients with osteoporosis, the effect of postoperative vertebral geometry reduction is better because of the larger space in the bone.

The primary cause of vertebral body compression fractures is the increase in stress on the vertebral body beyond its maximum bearing capacity. In addition to osteoporosis, other factors, such as existing geometric deformation of the vertebral body [29] and changes in the axis of gravity of the spine [27, 30], can also lead to vertebral body compression fractures. The results of this study indicate that PKP can significantly relieve low back pain and correct geometric deformities of the vertebral body in patients with normal BMD or osteopenia, although the effect is slightly worse than that of osteoporotic vertebral compression fractures.

Our study has some limitations, which should be mentioned. First, it was a retrospective analysis in a single center with a small sample size and short-term follow-up. Second, no previous studies have yet reported the same grouping method as ours, which may have caused subjective bias in the categorization process. Third, there were fewer comparative indicators during the follow-up period, and pain assessment was not performed using multidimensional scales, nor was there a relationship between the subjective feelings of patients and angle recovery. Therefore, future multicenter studies with larger sample sizes are warranted to validate our conclusions.

## Conclusions

This study shows PKP can significantly relieve pain and correct the geometric deformity of compression fractures in patients with normal BMD, osteopenia, and osteoporotic vertebral body compression fractures without significantly increasing the bone cement leakage rate. As such, PKP should be the first choice of treatment for osteoporotic vertebral compression fractures.

## Data Availability

Data used to support the findings of this study are available from the corresponding author upon request.

## Abbreviations

PKP: percutaneous kyphoplasty
BMD: bone mineral density
VAS: Visual Analogue Scale
WA: Wedged angle
KA: Kyphotic angle
